# Ethylene Antagonizes Salt-Induced Growth Retardation and Cell Death Process via Transcriptional Controlling of Ethylene-, BAG- and Senescence-Associated Genes in *Arabidopsis*

**DOI:** 10.3389/fpls.2016.00696

**Published:** 2016-05-19

**Authors:** Ya-Jie Pan, Ling Liu, Ying-Chao Lin, Yuan-Gang Zu, Lei-Peng Li, Zhong-Hua Tang

**Affiliations:** ^1^Key Laboratory of Plant Ecology, Northeast Forestry UniversityHarbin, China; ^2^Guizhou Academy of Tobacco ResearchGuiyang, China

**Keywords:** *Arabidopsis*, ethylene, salinity, cell death, BAG family gene

## Abstract

The existing question whether ethylene is involved in the modulation of salt-induced cell death to mediate plant salt tolerance is important for understanding the salt tolerance mechanisms. Here, we employed *Arabidopsis* plants to study the possible role of ethylene in salt-induced growth inhibition and programmed cell death (PCD) profiles. The root length, DNA ladder and cell death indicated by Evan's blue detection were measured by compared to the control or salt-stressed seedlings. Secondly, the protoplasts isolated from plant leaves and dyed with Annexin V-FITC were subjected to flow cytometric (FCM) assay. Our results showed that ethylene works effectively in seedling protoplasts, antagonizing salt-included root retardation and restraining cell death both in seedlings or protoplasts. Due to salinity, the entire or partial insensitivity of ethylene signaling resulted in an elevated levels of cell death in *ein2-5* and *ein3-1* plants and the event were amended in *ctr1-1* plants after salt treatment. The subsequent experiment with exogenous ACC further corroborated that ethylene could modulate salt-induced PCD process actively. Plant Bcl-2-associated athanogene (BAG) family genes are recently identified to play an extensive role in plant PCD processes ranging from growth, development to stress responses and even cell death. Our result showed that salinity alone significantly suppressed the transcripts of BAG6, BAG7 and addition of ACC in the saline solution could obviously re-activate *BAG6* and *BAG7* expressions, which might play a key role to inhibit the salt-induced cell death. In summary, our research implies that ethylene and salinity antagonistically control BAG family-, ethylene-, and senescence-related genes to alleviate the salt-induced cell death.

## Introduction

Salinity is one of the major abiotic stresses that adversely affect plant growth and crop productivity (Zhu, 2001; Flowers, 2004; Munns and Tester, 2008). Salt stress in plant cells is primarily caused by a combination of osmotic stress and salt-specific damage resulting from high Na+ concentration in the saline solution (James et al., 2006; Yang et al., 2013; Zhu et al., 2016). To antagonize salt-induced ionic toxicity, it has been largely documented that plants evolves lines of adaptive strategies to improve salt tolerance, mainly via minimizing Na+ buildup in photosynthetic organs (James et al., 2006) or retaining defined K+ level and K+/Na+ ratio in shoot (Agarie et al., 2007; Genc et al., 2007; Shabala, 2009). Shoot Na+ homeostasis of *Arabidopsis* plants grown in saline soils is conferred by reactive oxygen species (ROS) regulation of xylem-sap Na+ concentrations (Jiang et al., 2012). Simultaneously, osmotic and ionic stresses generate a cascade of secondary effect, including oxidative stress due to enhanced reactive oxygen species (ROS; Steffens and Sauter, 2009; Jiang et al., 2012; Yang et al., 2014), nutrient deficiency (Jiang et al., 2013), impaired photosynthetic system (James et al., 2006), and, consequently, cell death and growth reduction (Achard et al., 2006; Jha and Subramanian, 2014). Among various cues, salt-induced cell death has currently emerged as one of the most crucial components in plant salt responses (Affenzeller et al., 2009; Shabala, 2009; Andronis and Roubelakis-Angelakis, 2010; Hoang et al., 2015).

Programmed cell death (PCD) is one kind of physiological cell death and genetically determined process, present in both plant and animal cells (van Doorn and Woltering, 2004; Kabbage and Dickman, 2008; Steffens and Sauter, 2009). Recent studies have established that control of programmed cell death (PCD) pathways can be an important component that controls the outcome of a given stress response in plants (Li et al., 2016). At the physiological level, several morphological and biochemical similarities between apoptosis and plant PCD have been described, including DNA laddering, caspase-like proteolytic activity and cytochrome *c* release from mitochondria (Hoeberichts and Woltering, 2003; Andronis and Roubelakis-Angelakis, 2010). The earlier indicator of PCD before DNA fragmentation is the exposure of phosphatidylserine (PS) on the outer surface of plasma membrane, which can be monitored by flow cytometry (FCM) with a fluorescent conjugate of Annexin V-FITC (O'Brien et al., 1997; Pennell and Lamb, 1997; Halweg et al., 2005). PS exposure has been proved to be an earlier and preferential hallmark for plant PCD than DNA fragmentation measured by TUNEL assay (O'Brien et al., 1997). At the level of transcription, the plant *BAG* family genes have been identified to play an important role in plant PCD processes, which ranges from development, fungal resistance to abiotic stresses (Kabbage and Dickman, 2008; Fang et al., 2013; Li et al., 2016). Currently, seven members of the *Arabidopsis thaliana BAG* family genes have been identified (*AtBAG1-7*) and they might participate in plant PCD conferring plant tolerance to stresses (Fang et al., 2013). For example, the exogenous expression of *AtBAG4* in rice significantly improved salt tolerance at the whole plant level possibly through maintaining redox states (Hoang et al., 2015). More recent report showed that the *Arabidopsis bag6* knockout lines showed a more rapidly spreading cell death and higher sensitivity upon fungal pathogen (Li et al., 2016).

Plant PCD process is strictly mediated by hormone signals, such as ethylene, ABA or SA (Asai et al., 2000; Kim et al., 2010). Meanwhile, they often act in conjunction, like ABA and GA in barley aleurone layers, or SA and ethylene in HR-induced cell death (Hoeberichts and Woltering, 2003). Ethylene has been proven to be one of the positive regulators of PCD process during development under normal condition (Yamada and Marubashi, 2003; Bouchez et al., 2007). In *Arabidopsis*, ethylene stimulates the expression of senescence-associated genes (*ANACO29, ANACO92, RPK, SAG, ATG GBF*, and *GDH*) (Kim et al., 2015) and ethylene-related genes (*ERS2, ERF1, ACS2, ETR2*, and *EIN3*) (Fang et al., 2013). On the other hand, ethylene promotes the homeostasis of Na+/K+, nutrients and ROS to enhance plant tolerance to salinity (Tao et al., 2015). The salinity-induced ethylene signal is transduced mainly through the classical receptors-CTR1-EIN2-EIN3 pathway to regulate many effectors involved in plant growth and salinity response (Jiang et al., 2013; Yang et al., 2013; Li et al., 2015; Poór et al., 2015). Although ethylene alone is not sufficient to trigger PCD in plants, the cadmium- or camptothecin-induced cell death and the associated oxidative burst can be blocked by inhibition of ethylene signaling (de et al., 2002; Hoeberichts and Woltering, 2003; Yakimova et al., 2006). This evidence predicts the critical role of ethylene in mediating salt adaptation with respect to PCD pathway.

Regarding the ethylene signaling mutation, the loss of function of CTR1 generates *Arabidopsis* mutant *ctr1-1*, which shows constitutive ethylene responses (Chen et al., 2005). Downstream of EIN2, EIN3 transcription factor stimulate the expression of ethylene response target genes and the ethylene mutants *ein2-5* and *ein3-1* show entirely or partly ethylene insensitivity (Bleecker and Kende, 2000; Guo and Ecker, 2004). Here, the mutants affected in ethylene signaling together with wild-type Col-0 were employed to dissect the role of ethylene during salt-induced PCD process. The salt-stressed seedlings of various accessions are applied into comparison of the PCD processes through the assays of DNA fragmentation and PS exposure. Secondly, the wild-type plant protoplasts were isolated and directly treated with salinity to confirm the blocked action of exogenous ethylene releaser on the salt-induced PCD hallmarks. Further, the transcript abundance of 7 *BAG* family genes were analyzed and compared with that of ethylene-associated and senescence-related genes. Our results revealed that ethylene tightly antagonized the process of salt-induced cell death to confer plant salt tolerance.

## Materials and methods

### Plant materials and treatments

*Arabidopsis thaliana* mutants employed in this study were in the Col-0 background (WT), including *ein2-5, ein3-1* and *ctr1-1* mutants, kindly provided by Prof. Hong-Wei Guo from Peking University. Seeds were surface sterilized by 70% ethanol solution containing 1% Triton X-100. They were washed with ethanol and dried under sterile conditions.

#### Experiment 1

The sterilized seeds were then placed on agar plates including 0.8 fold MS salt, 1% sucrose, and pH 5.7, 0.6% Agar supplemented with 0, 50, 100, or 150 mM NaCl. All the plates were kept at 4°C in the dark for 4 d to synchronize germination, and then transferred to light at 23°C under a 16/8 light/dark cycle and a light intensity of approximately 220 mmol m^−2^ s^−1^ as described previously (Yang et al., 2013). After 14 d of germination, the root length and DNA ladder in NaCl-treated and control seedlings were measured (Jacyn Baker and Mock, 1994). Each treatment was replicated three times.

#### Experiment 2

The 7-d-old seedlings in the control medium were transferred to perlite/sand (1:1) irrigated daily with 1/2 strength Hoagland solution. After 7 d of transfer, Col-0 (WT) seedlings were collected from petri dishes after following treatments: for the measurements of PCD, Control (non-saline), 50 mM NaCl, 100 mM NaCl for 14 d; for the measurements of gene expression, Control (non-saline), 100 mM or 100 mM NaCl + 50 μM ACC NaCl for 14 d; for the measurements of protoplast PCD, Control (non-saline), 200 mM NaCl and 200 mM NaCl + 10 μM ACC, the leaves of Col-0 (WT) seedlings were bulked and used for isolation of protoplasts (Yang et al., 2014). Early PCD levels in protoplasts were measured at 10, 20, 30, 60, 90, 120, 150 min after treatments, respectively. The isolated protoplasts were simultaneously incubated in salt condition (200 mM NaCl) in the presence of 10 μM ACC, 10 μM DPI (inhibitor of NADPH oxidases, Sigma-Aldrich) or 10μM ACC + 5 mM H_2_O_2_. PCD profile was measured after 150 min of treatments. The DPI stock solution was prepared in 100% DMSO.

### Measurement of root length

Seedling images were taken from the bottom of the plates using a scanner (Epson, Suwa, Japan). Root length was measured using the Neuron J plugin (Meijering et al., 2004) for Image J (Schneider et al., 2012). Three replicates were performed and every replicate used 5 plants.

### DNA extraction and analysis

Additionally, an accepted biochemical criterion is the detection of an oligosomal DNA ladder the rungs of which are multiples of 180 bp. This ladder is due to a caspase-activated DNase that degrades DNA during apoptosis (Enari et al., 1998)*. Arabidopsis* genomic DNA was extracted according to the CTAB method. Leaf tissues were ground in liquid N_2_ immediately after being collected from the plants and then the frozen samples were homogenized in an extraction buffer that contained 2% CTAB, 100 mM Tris-HCl, 20 mM EDTA, pH 8.0 and 1.4 mmol NaCl, and the mix incubated at 65°C for 1 h. DNA electrophoresis was performed to assay DNA fragmentation. DNA samples of 5 μg DNA (per lane) were loaded on a 1.8% agarose gel at constant 70 V. The DNA was visualized by staining with 0.3 μg mL^−1^ ethidium bromide.

### Protoplasts preparation

Protoplasts were isolated according to the report (Halweg et al., 2005) and the protocol was modified for our specific application. The rosette leaves mentioned above were used to prepare protoplast and the leaf blade were cut into pieces of 0.5–1 cm^2^ and placed upside down without midribon in a sterile petri-dish with 10 mL of protoplast solution. The protoplast solution was composed of 0.4 M mannitol, 5 mM MES and 8 mM CaCl_2_, pH 5.6, 1% of cellulase R-10 (Yakult, Japan), 0.25% macerozyme R-10 (Yakult, Japan), and 0.03% Bovine serum albumin (Amresco, USA). Then the leaves were incubated for 4 h at 27°C in the dark. Subsequently, this mixture was passed through a sterile stainless steel mesh sieve (mesh size 100 μm). The filtered protoplast suspension was centrifuged for 5 min at low speed (600 rpm, 25°C). Intact protoplasts were collected from the interphase and transferred into a new tube. Then 10 mL protoplast washing buffer (5 mM 2-[N-morpholino] ethane-sulphonic acid, 8 mM CaCl_2_, 0.4 M mannitol, pH 5.6) were added and mixed gently followed by a second centrifugation under the same conditions. This washing step was repeated and a small aliquot of the washed protoplasts was used for the estimation of protoplast density in a hematocytometer. Before assay of PCD, the protoplast density was adjusted to 3–5 × 10 ^5^ mL^−1^. Three replicates were performed and every replicate used 5 plants.

### Observation and assessment of protoplast PCD

The protoplast PCD occurrence was observed by laser scanning confocal microscope (LSCM, Nikon C1Plus, Japan) and quantitative assessment of percentage of PCD-occurring protoplasts to the normal ones was performed with flow cytometry (FCM). Briefly, 5 × 10^5^ protoplasts mL^−1^ were washed twice with protoplast washing buffer, resuspended cells in 195 μL prediluted binding buffer and added 5 μL Annexin V-FITC kit (Bender MedSystems, USA), mixed and incubated for 10 min at room temperature (Solution 1). The solution 1 was firstly observed and photographed using LSCM with 488 nm band-pass excitation and 530 nm band-pass emissions.

Protoplasts in the identical volume of the Solution 1 were re-suspended in 190 μL prediluted binding buffer, added 10 μL of the 20 μg mL^−1^ propidium iodide (PI) stock solution. After addition of another 300 μL binding buffer, the suspended protoplasts were analyzed by flow cytometer PAS (Partec GmbH, Bioflow, Martinsried, Germany) with 488 nm band-pass excitation and 530 nm band-pass emission. The events of PCD occurrence was examined according the rules (a) The FITC^−^/PI^−^ population indicate intact cells; (b) the FITC^+^/PI^−^ population indicate early PCD protoplasts; (c) the FITC^+^/PI^+^ population indicate late PCD cells. Three independent sets of experiments were performed with 10,000 protoplasts approximately and the averages were presented. Three replicates were performed and every replicate used 5 plants.

## RNA isolation and quantitative real time RT-PCR analysis

The qPCR assay has been designed according to the Minimum Information for Publication of Quantitative Real-Time PCR Experiments (MIQE) guidelines (Table S2; Bustin, 2010; Remans et al., 2014). We treated the Col-0 (WT) seedlings to study the effects on expression of BAG family genes, ethylene-related genes and senescence-related genes in response to the treatments with salinity and ACC. Seedlings after treatments were collected and ground in liquid nitrogen. Total RNA was extracted by TRIZOL reagent (Invitrogen). DNA contamination was removed by Dnase I following the instructions provided by the manufacturer (TaKaRa, Japan). RNA purity was observed using 1% agarose gel electrophoresis and RNA concentration was determined using a Nanodrop spectrophotometer (Thermo). The cDNA was synthesized from total RNA (2 ug) using ReverTra Ace QPCR RT Kit (Toyobo, Japan) according to the manufacturer instructions, using oligo (dT20) as the primer.

In our experiment, *ACTIN2, PP2A*, and *UBC* were used as candidate reference genes to evaluate their suitability based on a single melting temperature (Tm) and a single band on a 10% polyacrylamide gel (Figures S1–S3). According to melting curves and electrophoregram, we choose *ACTIN2* gene as reference gene. All primers have been described and optimized previously in accordance with the MIQE guidelines. The qRT-PCR analysis with cDNA as template and the expression of target genes as well as reference gene was monitored by quantitative real-time PCR using appropriate transcripts and primers (Table S1) were performed using a SYBR Premix Ex Taq (TaKaRa, Japan) with an initial denaturation at 95°C for 30 s, followed by 35 cycles at 94°C for 30 s, 56°C for 30 s and 72°C for 30 s. Experiments were repeated three times for each sample as technical replicates to ensure the reproducibility of results. The relative expression value was calculated using the 2-ΔΔCt method.

### Statistical analysis

Principal component analysis (PCA), the multivariate analyze tool, is used to reduce a set of original variabilities and to extract a small number of latent factors (principal components [PCs]) for analyzing relationships among the observed variabilities. PCA was performed in order to investigate the distribution pattern of quantitative gene expression in response to ACC and NaCl in *Arabidopsis* seedlings. Statistical treatment was carried out by analysis of variance (ANOVA) and PCA using SPSS-17 statistical software package. The two-way ANOVA was performed to examine the effects of salinity and genetic mutation on root length, respectively or jointly. Results were represented as the means ± standard error (S.E.). Data were analyzed using one-way analysis of variance and Duncan multiple range tests (*P* < 0.05; Pan et al., 2015).

## Results

### The phenotypic changes and leaf DNA fragmentation induced by salinity

**Figure 1:**
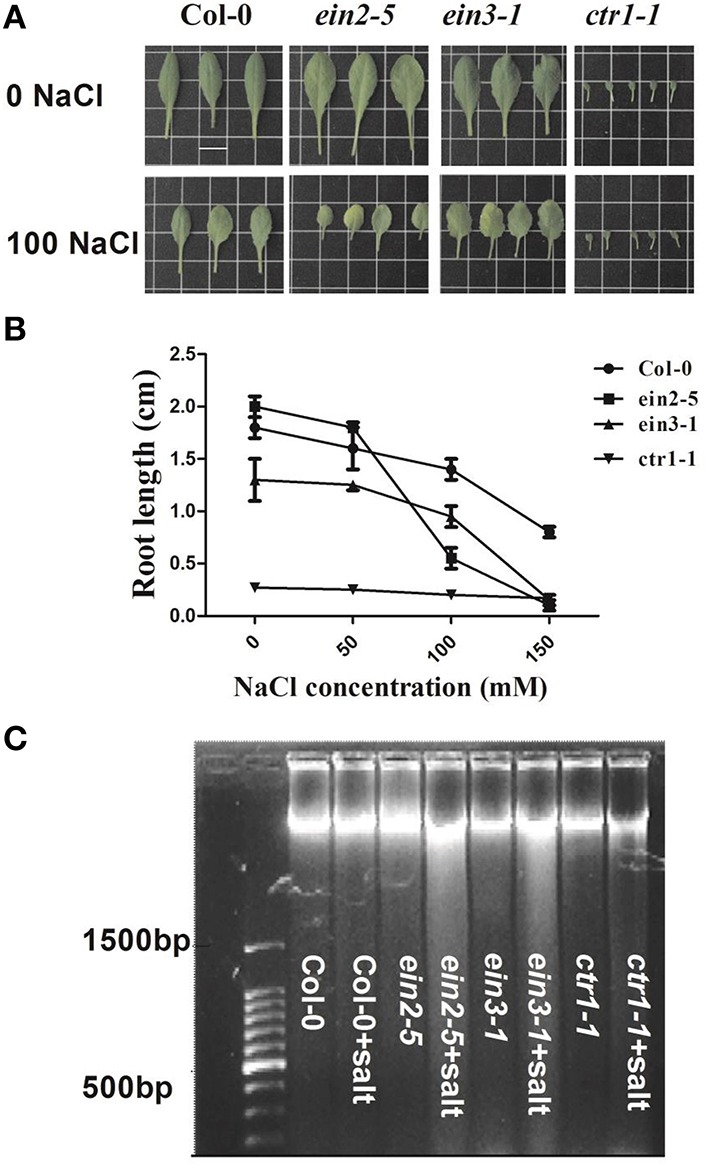
**PCD occurrence among various accessions of ***Arabidopsis*** plants after 14-day treatment**. Seedlings of Col-0, *ein2-5, ein3-1*, and *ctr1-1* were treated with 0, 50, 100, and 150 mM NaCl contained in nutrient solution for 14 days. **(A)** Plants of above-mentioned four genotypic seedlings treated with 0 or 100 mM NaCl. Scale bars, 1 cm. **(B)** Root length reduction induced by salinity in four genotypic plants. Values are mean ± SE from 5 seedlings per replicate (*n* = 5 replicates). The different letters at the top of each bar indicate the significant difference among various treatments (*P* < 0.05). **(C)** DNA ladder when plants were treated with 0 mM or 100 mM NaCl.

The PCD events among various lines of plants were observed by root elongation measurement and DNA laddering after treatment of salinity. Figure 1A showed the leaf morphological changes of seedlings under 0 or 100 mM NaCl. The plants of *ein2-5* and *ein3-1* displayed reduction of leaf area and salt-induced senescence symptoms obviously compared to their controls. However, Col-0 or *ctr1-1* plants were not largely affected by the applied salinity as the contrast of *ein2-5* or *ein3-1*. Root elongation measurement showed that *ein2-5* mutant plants were the most hypersensitive to NaCl (Figure 1B). The concentration of NaCl was estimated as I_50_, which decreased the root growth rate by 50 % compared to control medium (without NaCl). The I_50_ concentrations for *ein2-5* seedlings were 100 mM, with the root of *ein2-5* and *ein3-1* entirely restrained under 150 mM NaCl condition. The root elongation of Col-0 or *ctr1-1* plants was observed to be 40–50% shorter especially upon 150 mM NaCl medium. These results showed that insensitivity of ethylene signaling exaggerated plant sensitivity to salinity in light of growth status and root elongation. DNA laddering experiment indicated that salinity induced obvious DNA fragmentation in *ein2-5* and *ein3-1* plants. However, this process did not obviously appear in salt-stressed Col-0 or *ctr1-1* plants (Figure 1C). The two-way ANOVA analysis showed that salinity and genetic lines significantly affected root length (*F*_salinity_ = 295.75; *F*_ethylene_ = 332.46; *F*_S ∗ E_ = 48.99).

### The *in vivo* protoplast PCD occurrence induced by salinity

**Figure 2:**
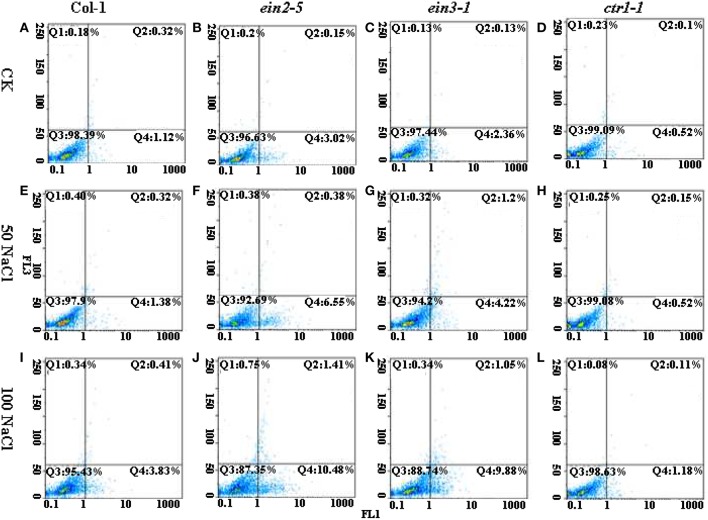
**Flow cytometric pictures of PCD occurrence among four lines of ***Arabidopsis*** plants under the control or saline conditions**. Seedlings of Col-0, *ein2-5, ein3-1*, and *ctr1-1* were treated with 0, 50, or 100 mM NaCl. The protoplasts were stained with AnnexinV (FL1 channel) and PI (FL2 channel), and then analyzed by FCM. The Q4 Zone indicates early PCD cells; The Q2 Zone indicates late PCD cells; The Q3 Zone indicates intact cells. **(A)** The seedling of Col-0 were treated with 0 mM NaCl. **(B)** The seedling of *ein2-5* were treated with 0 mM NaCl. **(C)** The seedling of *ein3-1* were treated with 0 mM NaCl. **(D)** The seedling of *ctr1-1* were treated with 0 mM NaCl. **(E)** The seedling of Col-0 were treated with 50 mM NaCl. **(F)** The seedling of *ein2-5* were treated with 50 mM NaCl. **(G)** The seedling of *ein3-1* were treated with 50 mM NaCl. **(H)** The seedling of *ctr1-1* were treated with 50 mM NaCl. **(I)** The seedling of Col-0 were treated with 100 mM NaCl. **(J)** The seedling of *ein2-5* were treated with 100 mM NaCl. **(K)** The seedling of *ein3-1* were treated with 100 mM NaCl. **(L)** The seedling of *ctr1-1* were treated with 100 mM NaCl.

The PCD events among various lines of plants were observed by FCM measurement after treatment of salinity. FCM assay of PCD occurrence showed that *ein2-5* and *ein3-1* mutant plants significantly delayed PCD occurrence under non-saline condition compared to the condition of Col-0 (Figures 2A–C). In contrast, salinity treatment for 14 d led to a continuous increase of PCD occurrence in Col-0, *ein2-5* and *ein3-1* plants compared to their controls (Figures 2E–G). The statistic results of early and late PCD occurrence among *Arabidopsis* mutants under control and saline conditions were showed in Figure 2. The data indicated that salt stress-induced PCD was greatly promoted in *ein2-5* and *ein3-1*. The ratios of early PCD cells to normal ones were both more than 5% with 50 mM NaCl treatment (Figures 2F,G), and under 100 mM NaCl, the percentage of early PCD cells increased to 11.08%, 9.16% in *ein2-5* and *ein3-1* (Figures 2J,K), respectively. In contrast, the early PCD cells ratio were not largely changed in Col-0 and *ctr1-1* mutant under salt stress. The percentage of early PCD cells was no more than 4% under salt stress (Figures 2E,H). In addition, the ratios of late PCD cells of *ein2-5* and *ein3-1* changed significantly between the concentration of 50 mM and 100 mM NaCl (Figures 2F,G,J,K). There was no significant difference between Col-0 and *ctr1-1* with different concentrations of NaCl treatment (Figures 2E,H,I,L). These results showed that ethylene insensitive mutants were more susceptibly affected by salt stress and their PCD processes were accelerated by NaCl treatment.

By summarized of Pearson' s regression coefficients (R) and *P*-value between PCD processes and increased saline concentrations for individual *Arabidopsis* accessions (Table 1), the results showed that the processes of early PCD in all accessions were tightly correlated with increased salt stress. Especially, the coefficients R for *ein2-5 and ein3-1* plants were 0.97 and 0.91, much higher than those in Col-0 and *ctr1-1* plants. The survey of correlations between late PCD and salt stress displayed that the significant correlation (*R* = 0.809, *P* = 0.01) was observed in *ein2-5* mutant plants only. The mutations such as ethylene insensitive mutant *ein2-5 and ein3-1* are more sensitive to salt stress than the wild type plants, and it was suggested that mutations could largely alter PCD processes in ethylene signaling pathway.

**Table 1:** **Regression analysis of early or late protoplast PCD occurrence of Col-0, ***ein2-5, ein3-1***, and ***ctr1-1*** plants in response to 0, 50, and 100 mM NaCl concentration respectively**.

**Parameters**	**Mutants**	**Salt concentrations**	
		***R***	***P*-value**
Early PCD	Col-0	0.869^**^	0.00
	*ein2-5*	0.972^**^	0.00
	*ein3-1*	0.913^**^	0.00
	*ctr1-1*	0.766^*^	0.02
	Total	0.556^**^	0.00
Late PCD	Col-0	−0.18	0.65
	*ein2-5*	0.809^**^	0.01
	*ein3-1*	0.51	0.16
	*ctr1-1*	0.09	0.83
	Total	0.380^*^	0.02

* Correlation significant at the 0.05 level (2-tailed);

** *Correlation significant at the 0.01 level (2-tailed)*.

### The *in vitro* protoplasts PCD affected by salinity and exogenous ACC

**Figure 3:**
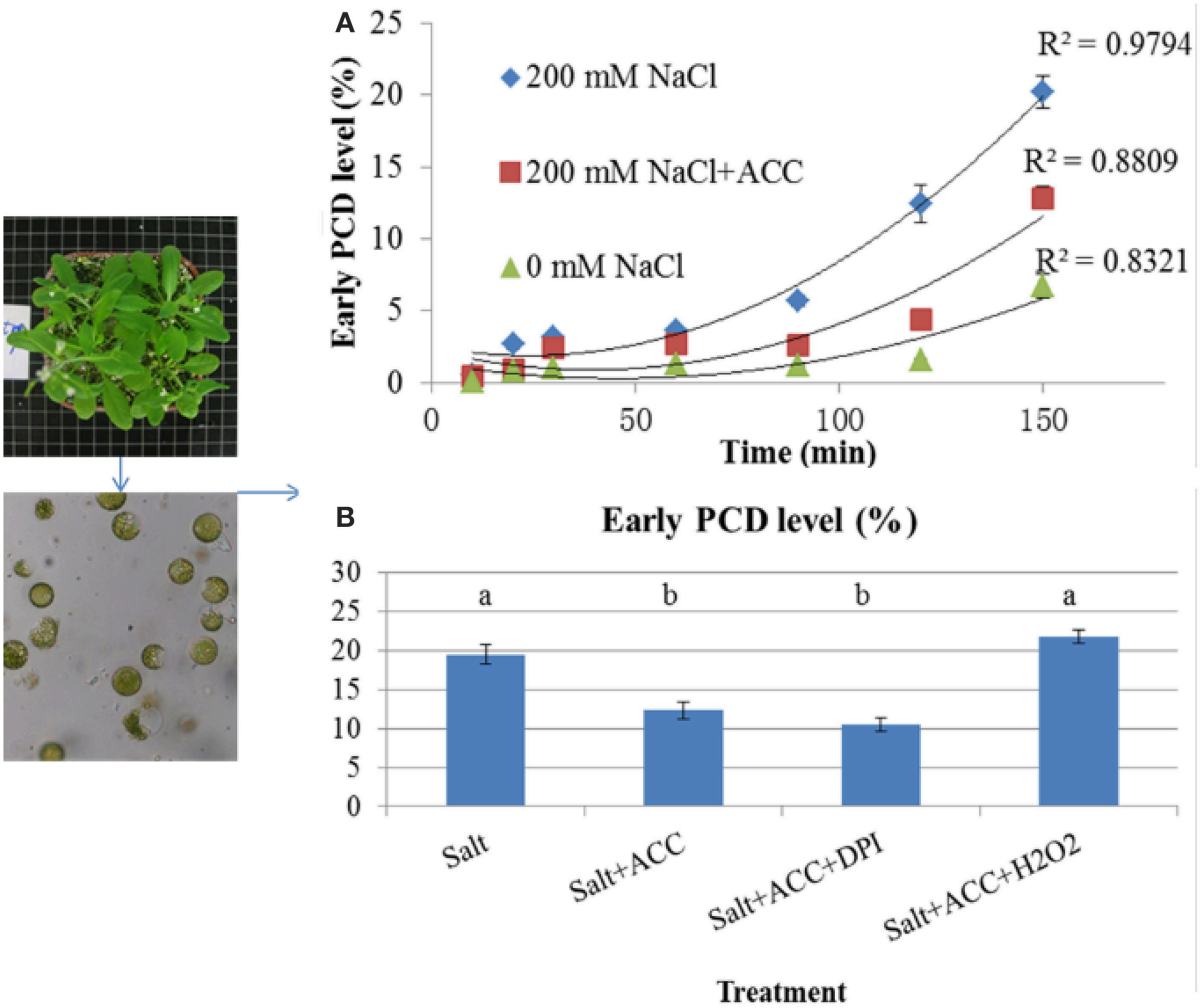
**The salt-induced PCD processes in isolated protoplasts with treatment by exogenous application of ACC and H_2_O_2_. (A)** The isolated protoplasts from the control Col-0 seedlings were treated with 0 mM NaCl, 200 mM NaCl, or 200 mM NaCl + 10 μM ACC; **(B)** The isolated protoplasts were simultaneously incubated in salinized condition (200 mM NaCl) in the presence of 10 μM ACC, 10 μM ACC+10 μM DPI, or 10 μM ACC+5 mM H_2_O_2_. PCD was measured after 150 min of the treatments. The results are shown the mean ± SE of three replicates and 5 plants for each replicate. The different letters denote the significant difference among different treatments (*P* < 0.05).

To testify the role of ethylene in amending salinity-PCD processes, the protoplasts were isolated from Col-0 and incubated in 0 mM NaCl, 200 mM NaCl, and 200 mM NaCl + 10 μM ACC solution (Figure 3). The PCD level was measured at 10, 20, 30, 60, 90, 120, 150 min, respectively (Figure 3A). As it shown in Figure 3A, the occurring rate of the protoplast PCD increased with time under such conditions. Additionally, the salinity of 200 mM NaCl applied to the protoplasts generated a rapid PCD-increasing rate compared to the events exhibited in the control solution. The application of ACC to the saline solution largely reduced the occurring rate of PCD in contrast to those without ACC.

In order to investigate the effects of redox status in the cells during PCD procedure, the isolated protoplasts were simultaneously incubated to 200 mM NaCl and 200 mM NaCl + 10 μM ACC in the presence of H_2_O_2_ or DPI (Figure 3B). After 150 min of treatment, PCD profiles were measured. The results suggested that the inhibition of salt-induced PCD by ACC was disrupted by exogenous application of H_2_O_2_ but retained by DPI (Figure 3B).

### Relative expressions of the PCD-, ethylene-, and senescence-related genes affected by salinity and exogenous ACC

**Figure 4:**
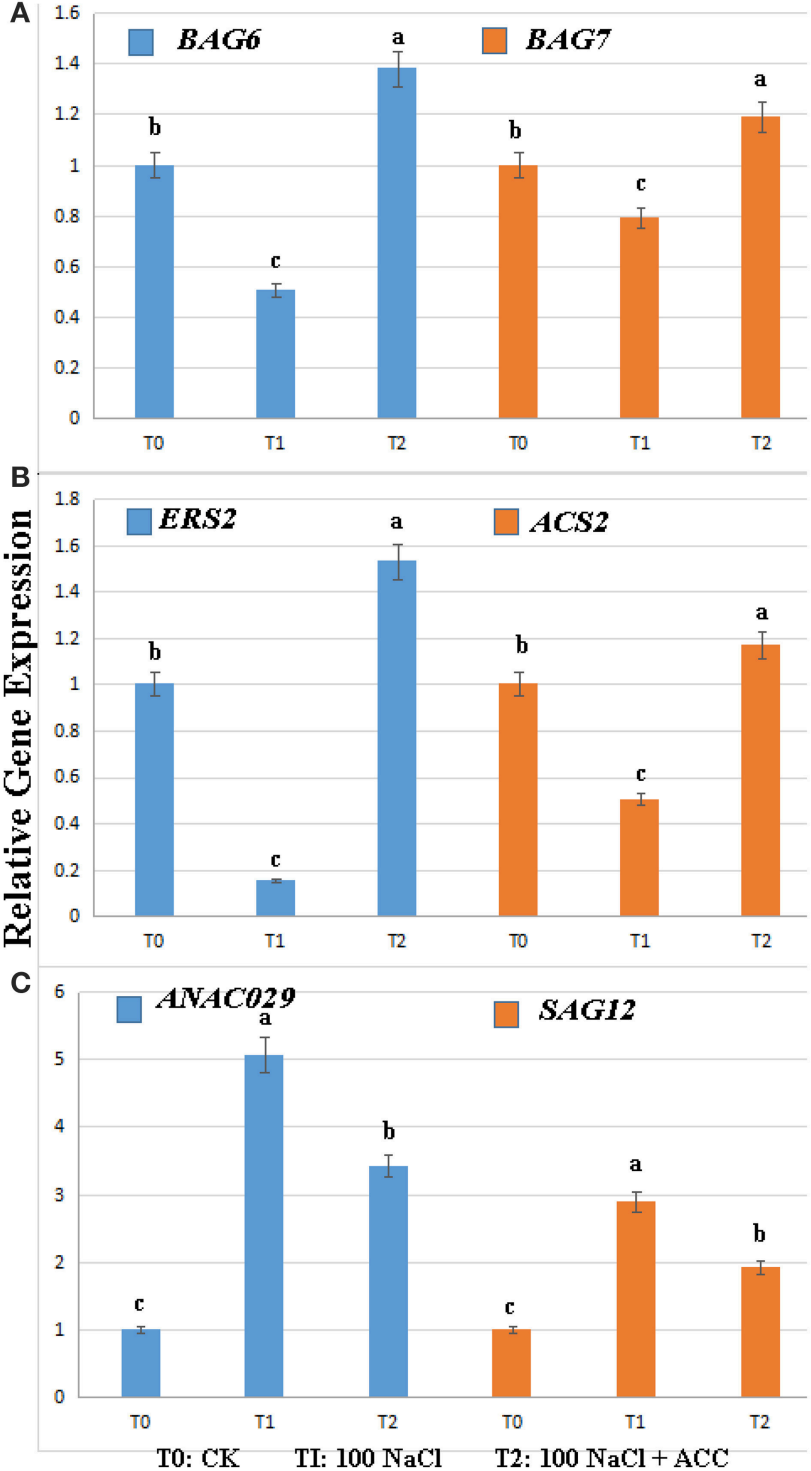
**Relative gene expression levels in the Col-0 plants upon salinity alone or plus ACC**. The results were analyzed using the comparative Ct method. **(A)** The expression of *AtBAG6 and AtBAG7* genes; **(B)** The expression of *ERS2 and ACS2* genes; **(C)** The expression of *ANAO029 and SAG12* genes; Treatment with 0 mM NaCl (T0), 100 mM NaCl (T1), 100 mM NaCl + 50 μM ACC (T2). Data were analyzed using one-way analysis of variance and Duncan multiple range tests. The different letters denote the significant difference among different treatments (*P* < 0.05). Each relative gene expression represents the average of three measurements, with error bars representing Standard Errors.

To reveal the molecular mechanism of ethylene controlling the salinity-induced cell death, we compared the expression levels of BAG family, ethylene-, and senescence-related genes in the wild-type plants treated with 0, 100 mM NaCl, 100 mM NaCl plus 50 μM ACC (Figure 4). The results showed that the treatment with salinity alone significantly suppressed the transcripts of *BAG6, BAG7, ERS2, and ACS2* (Figures 4A,B). However, the subsequent inclusion of ACC in the saline solution effectively reversed this inhibition of their expressions. The senescence-related genes under the above condition were then checked and the transcriptional abundance of *ANAC029* and *SAG12* was greatly triggered by salinity. Whereas, the addition of ACC in the saline solution inhibited this senescence process induced by salinity (Figure 4C). The above transcriptional data suggested that the PCD-, ethylene- and senescence-related genes actively participated in the salt-induced PCD process.

### The heat map analysis of total gene profiles in the wild-type plants upon salinity plus ACC

**Figure 5:**
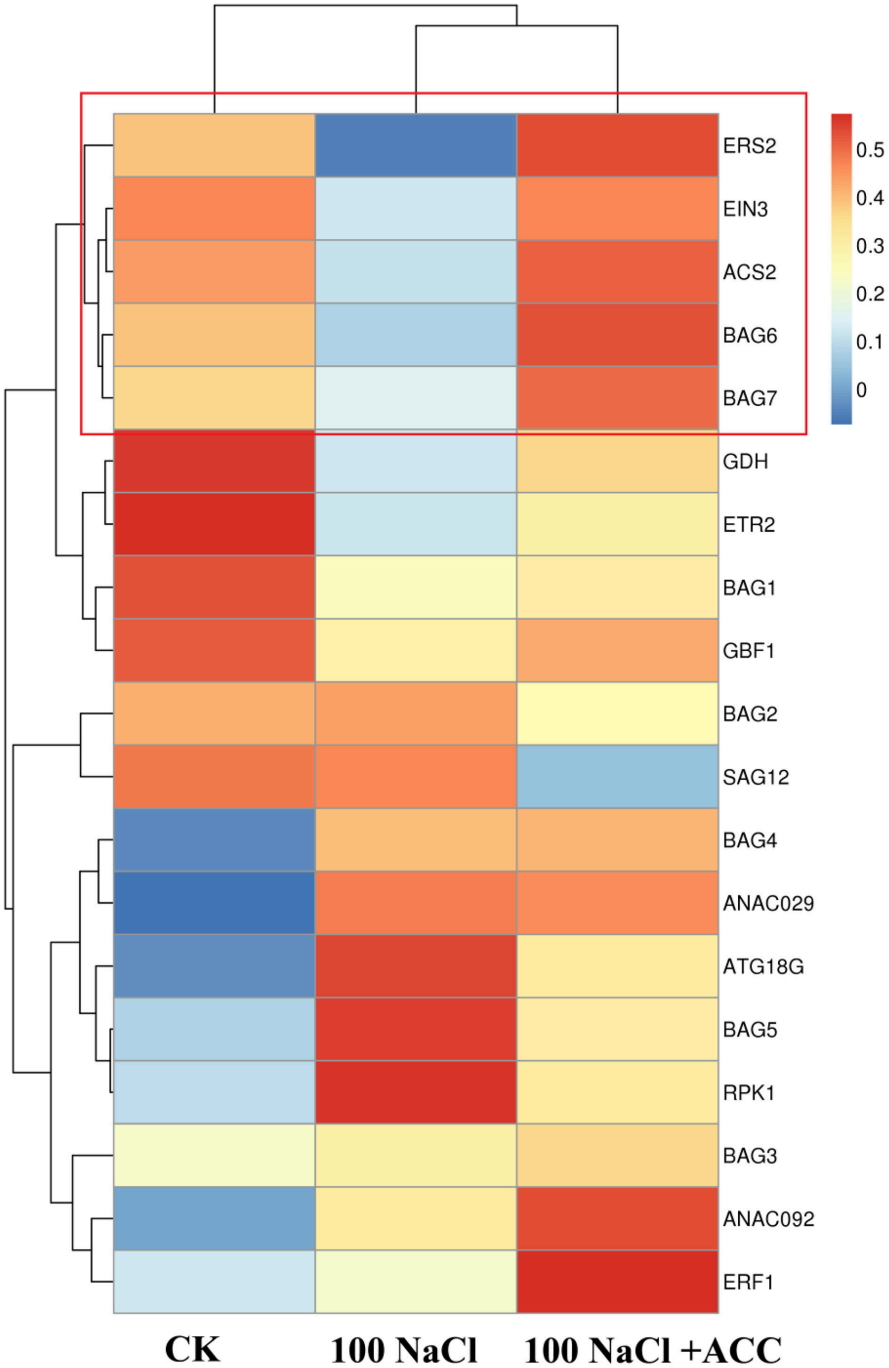
**The Heat Map analysis of gene expression mode in the Col-0 plants upon salinity alone or plus ACC**. The Heat Map shows the expression of *BAG* family genes, ethylene-related genes and senescence-related genes upon induction by NaCl and ACC. Treatment with 0 mM NaCl (Col-0), 100 mM NaCl, 100 mM NaCl + 50 μM ACC. The average linkage hierarchical clustering with Pearson correlation was used. Blue and red denote relative down-regulation and up-regulation, respectively.

We then profiling the expression mode of 19 hallmark genes involved the PCD-, ethylene- and senescence-related processes in the Col-0 plants upon salinity alone or plus ACC (Figure 5). The hierarchical clustering of different treatments found that the control plants shared a common gene expression mode with those upon salinity plus ACC. This indicated that ACC could effectively reverse the salinity-induced transcriptional responses. In addition, the expression modes of *EIN3, ACS2, BAG6, BAG7*, and *ERS2* were in clustered common indicating their tight interaction during these treatments. On the contrast, salinity stress significantly promoted gene expression of *ANAC029* and *SAG12*, and these senescence genes were suppressed when ACC was added in the saline solution. Whereas, other investigated genes, such as *BAG1-BAG5*, did not displayed a consistent response as expected, displaying a diverse expression mode.

## Discussions

Ethylene has been reported to be involved in control of salt-induced ROS burst, ionic imbalance and osmotic stress to confer plant salt resistance (Jiang et al., 2013; Lin et al., 2013; Yang et al., 2013; Peng et al., 2014; Tao et al., 2015). Generally, promotion of ethylene biosynthesis and signal transduction could enhance plant tolerance to salinity, while inhibition of it leads to increased plant sensitivity to salinity (Tao et al., 2015). The salt-induced K+ deprivation and ROS burst will further activate cell death in plants (Huh et al., 2002; Affenzeller et al., 2009; Shabala, 2009). Here, we provided evidence to the critical role of ethylene controlling the salt-induced cell death process to confer salt tolerance in *Arabidopsis*.

The phenotypic changes of four lines of *Arabidopsis* were firstly compared under non-saline and saline conditions. Under control condition, the phenotipic responses of *ein2-5* and *ein3-1* resulted in an increment in leaf area and root lenght and on the other hand a constrained growth in *ctr1-1* compared to the Col-0 plants. It was proposed that smaller plants may be less vulnerable to stress because they have less surface area (Achard et al., 2006; Yang et al., 2013). The decreased leaf area pertain to a low transpiring rate, which help plants avoid excess Na+ intake, Na+ xylem loading into shoot and then Na+ over accumulation (Jiang et al., 2013; Tao et al., 2015; Zhu et al., 2016). For example, the *ctr1-1* plants were reported to display a reduced increase in shoot Na+ concentration compared with wild-type plants following salinity treatment and maintain a relatively high shoot K+ concentration in both control and salinity treatment conditions (Jiang et al., 2013). This might be a critical physiological mechanism for ethylene role in salt resistance, because gain-of-function of ethylene biosynthesis or signaling normally generates smaller plant size. Our observations confirmed that the *ctr1-1* plants displayed a slight reduction in leaf area and root elongation, while, *ein2-5* or *ein3-1* ones showed a magnified retardation in plant growth compared to the wild-type plants under a saline condition (Figures 1A,B).

The salt-induced cell death occurrence then was compared among the four lines of plants. The salt-induced cell death acceleration in plants was generally considered as a key indicator of salt damage symptom (Dickman et al., 2001; Shabala, 2009; Jha and Subramanian, 2014; Yang et al., 2014). The recent evidence showed that exogenous expression of anti-apoptotic genes *AtBAG4* (*Arabidopsis*) significantly improves salinity tolerance in rice at the whole plant level (Hoang et al., 2015). Therefore, the ability to minimize cell death process induced by salinity is of importance for plant salt tolerance. Our DNA laddering experiment revealed that the salt-induced DNA fragmentation, one of the hallmarks of cell death, was accelerated in *ein2-5* and *ein3-1* leaves, whereas, inhibited in the Col-0 or *ctr1-1* (Figure 1C). In leaf protoplast, the exposure of phosphatidylserine (PS) on the outer surface of plasma membrane was detected by FCM, which is the hallmark of PCD, suggesting that loss-of-function of ethylene signaling resulted in decreased plant ability to inhibit the occurrence of salt-induced PCD (Figure 2). This FCM method was previously applied to quantitatively detect animal and plant cells undergoing PCD (O'Brien et al., 1997; Halweg et al., 2005).

These observations showed that ethylene signaling was helpful for plants to decrease salt-induced cell death both at the whole plant or cell levels. This action is consistent with the recent research that Silicon mitigates cell death in cultured tobacco BY-2 cells subjected to salinity relying on ethylene emission (Liang et al., 2015). Our time curve of *in vitro* protoplast PCD occurrence also confirmed that exogenous ACC effectively delayed the salt-induced PCD and this effect of ACC could be reversed by simultaneous addition of H_2_O_2_ (Figure 3). The excess accumulation of ROS is thought to be key inducers of PCD in plants under stressed conditions (Shabala, 2009; Jha and Subramanian, 2014). Our results indicated that ethylene might control the salt-induced cell death depending on redox states regulation. Large body of report illustrated that ethylene could deter the salt-induced ROS to confer plant salt tolerance (Munns and Tester, 2008; Lin et al., 2013; Peng et al., 2014; Poór et al., 2015; Tao et al., 2015).

At last, we compared the expression levels of *BAG* family, ethylene-, and senescence-related genes in the wild-type plants subjected to salinity alone or plus ACC. Interestingly, salinity alone significantly suppressed the transcripts of *BAG6, BAG7, ERS2, and ACS2* (Figure 4). Plant *BAG* genes are tightly involved in environmental responses and they block several biotic and abiotic cell death-mediated processes while conferring cyto-protection under sevaral adverse condition and during plant development (Dickman et al., 2001; Kabbage and Dickman, 2008; Williams et al., 2010; Li et al., 2016). For example, *BAG6* was required in resistance to fungal-induced cell death in *Arabidopsis* (Li et al., 2016) and the deletion of BAG7 compromises heat and cold tolerance (Williams et al., 2010). Our results showed that the inclusion of ACC in the saline solution could obviously re-activate *BAG6* and *BAG7* expressions, which might play a key role to inhibit the salt-induced cell death as reported before. The Heat Map analysis of total gene profiles in our study found that *EIN3, ACS2*, and *ERS2* were in clustered common with *BAG6* and *BAG7* (Figure 5). In addition, SAG12, an *Arabidopsis* gene encoding a cysteine protease, which is expressed only in senescent tissues (Noh and Amasino, 1999), is specifically activated by developmentally controlled senescence pathways, in our study, as well as than the abundance of *ANAC029* was also greatly triggered by salinity and reversed by addition of ACC. These results confirmed the essential role of ethylene biosynthesis and signal transduction in plant salt tolerance (Peng et al., 2014; Tao et al., 2015).

Our results provided evidence that the salt-induced PCD process was commonly antagonized by ethylene signaling. The altered salt-induced PCD level mediated by ethylene was proposed to contribute to an improved salt sensitivity in *ein2-5* and *ein3-1* plants and increased salt tolerance in *ctr1-1*. When salinity was directly applied to *Arabidopsis* protoplasts, the external addition of ethylene precursor ACC mostly inhibited salt-induced PCD process. This phenomenon suggests that the pathway of PCD is involved in alteration of salt response which is regulated by ethylene. At the transcriptional level, we compared the expression levels of BAG family, ethylene-, and senescence-related genes in the wild-type plants subjected to salinity alone or plus ACC. Interestingly, salinity alone significantly suppressed the transcripts of *BAG6, BAG7, ERS2*, and *ACS2* and addition of ACC in the saline solution could obviously re-activate *BAG6* and *BAG7* expressions, which might play a key role to inhibit the salt-induced cell death. Based on our findings, we propose that ethylene and salinity antagonistically modulate the salt-induced cell death via controlling the transcripts of BAG family, ethylene-, and senescence-related genes in *Arabidopsis*. The complex mechanisms need to be intensively investigated in the future.

## Author contributions

Conceived and designed the experiments: YP, ZT, YZ. Performed the experiments: YP, LL. Analyzed the data: YP, YL. Wrote the paper: YP, LPL.

### Conflict of interest statement

The authors declare that the research was conducted in the absence of any commercial or financial relationships that could be construed as a potential conflict of interest.

## Abbreviations

ACC: 1-aminocyclopropane-1-carboxylic acid
*ACS2*: 1-aminocyclopropane 1-carboxylate (ACC) synthase 2
*BAG*: Bcl-2-associated gene
CTAB: Cetyltrimethyl ammonium bromide
*CTR*: Constitutive triple response
DPI: Diphenylene iodonium
*EBF*: *EIN3*-binding F-box
*EIN2*: Ethylene insensitive 2
*EIN3*: Ethylene insensitive 3
*ERF1*: Ethylene response factor 1
*ERS2*: Ethylene response sensor 2
*ETR2*: Ethylene receptor 2
FCM: Flow cytometry
*GBF*: G box binding factor
*GDH*: Glutamate dehydrogenase isoenzyme
PCA: Principal component analysis
PCD: Programmed cell death
PS: phosphatidylserine
*RPK*: Receptor-like protein kinase
*SAG*: Senescence associated genes.
