# Supplementary figures and images for: Ethylene Antagonizes Salt-Induced Growth Retardation and Cell Death Process via Transcriptional Controlling of Ethylene-, BAG- and Senescence-Associated Genes in Arabidopsis
Source: Front Plant Sci. 2016 May 19;7:696. doi: 10.3389/fpls.2016.00696 (PMC4872043; doi:10.3389/fpls.2016.00696)

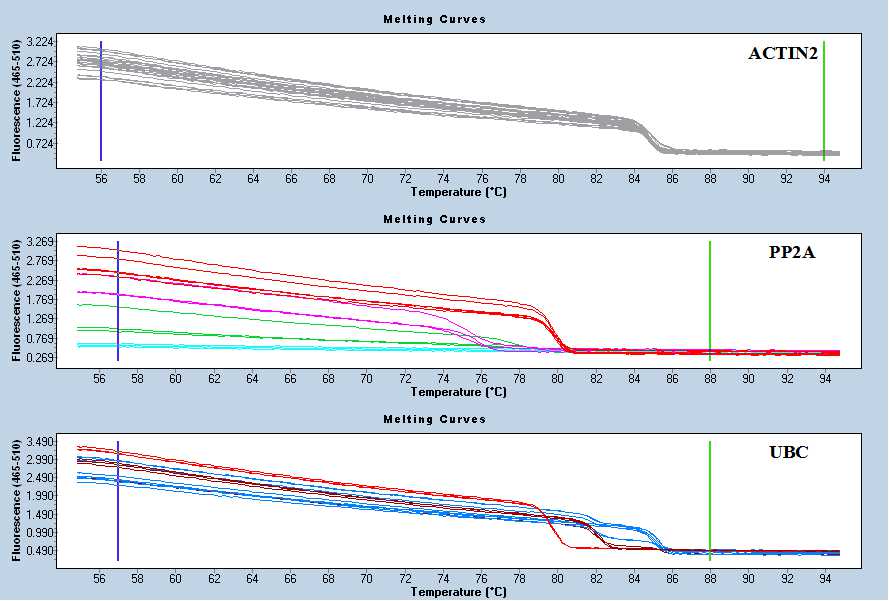

Supplement: Supplementary file 3 [file Image1.JPEG]

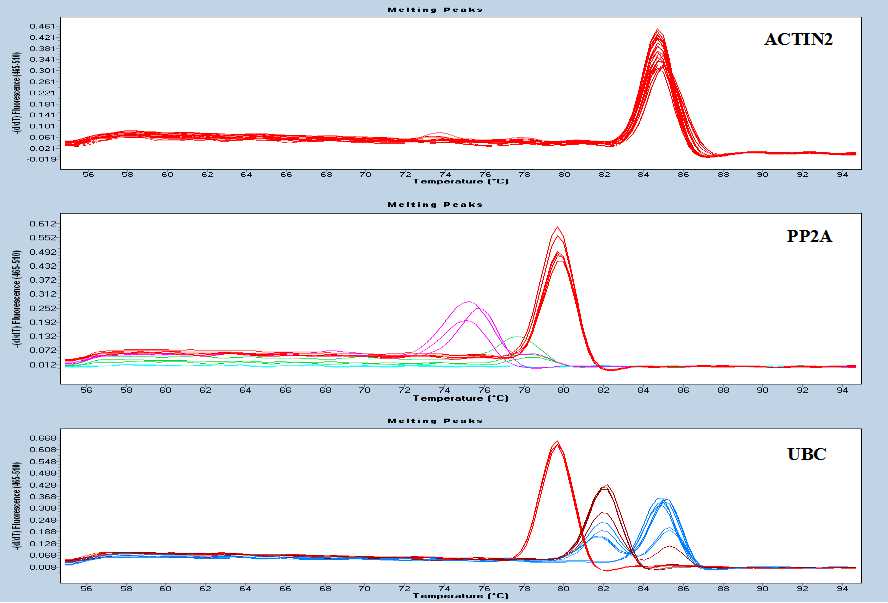

Supplement: Supplementary file 4 [file Image2.JPEG]

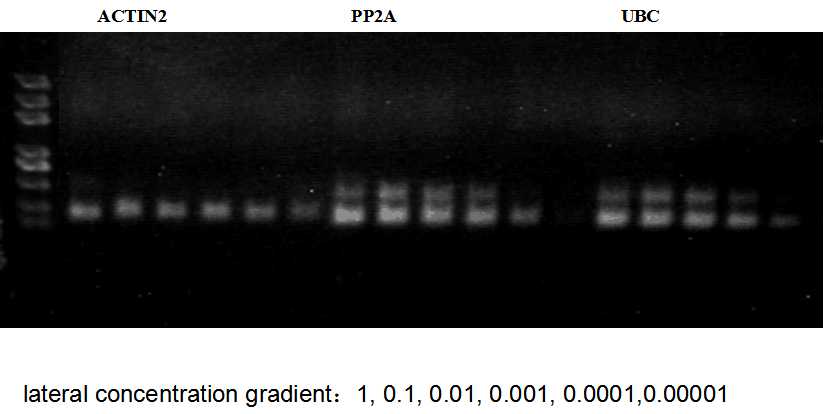

Supplement: Supplementary file 5 [file Image3.JPEG]
